# The Effects of Virgin Coconut Oil on Bone Oxidative Status in Ovariectomised Rat

**DOI:** 10.1155/2012/525079

**Published:** 2012-08-15

**Authors:** Mouna Abdelrahman Abujazia, Norliza Muhammad, Ahmad Nazrun Shuid, Ima Nirwana Soelaiman

**Affiliations:** Department of Pharmacology, Faculty of Medicine, Universiti Kebangsaan Malaysia, Jalan Raja Muda Abdul Aziz, 50300 Kuala Lumpur, Malaysia

## Abstract

Virgin coconut oil (VCO) was found to have antioxidant property due to its high polyphenol content. The aim of this study was to investigate the effect of the virgin coconut oil on lipid peroxidation in the bone of an osteoporotic rat model. Normal female Sprague-Dawley rats aged 3 months old were randomly divided into 4 groups, with 8 rats in each group: baseline, sham, ovariectomised (OVX) control group, and OVX given 8% VCO in the diet for six weeks. The oxidative status of the bone was assessed by measuring the index of lipid peroxidation, which is malondialdehyde (MDA) concentration, as well as the endogenous antioxidant enzymes glutathione peroxidase (GPX) and superoxide dismutase (SOD) in the tibia at the end of the study. The results showed that there was a significant decrease in MDA levels in the OVX-VCO group compared to control group. Ovariectomised rats treated with VCO also had significantly higher GPX concentration. The SOD level seemed to be increased in the OVX-VCO group compared to OVX-control group. In conclusion, VCO prevented lipid peroxidation and increased the antioxidant enzymes in the osteoporotic rat model.

## 1. Introduction

Osteoporosis is a chronic systemic skeletal disease characterized by a low bone mass and loss of bone tissue and micro-architecture. The bone becomes weak and fragile with a consequent increase in the fracture incidence [1]. According to World Health Organization (WHO), the osteoporosis is defined as having a bone mineral density (BMD) of 2.5 standard deviations below the mean for young healthy adults of the same gender or below the peak adult bone mass (*T*-score). Postmenopausal women who have *T*-scores of less than 1 SD below the mean are considered as having a low bone density and this places them at an increased risk of osteoporosis [2].

Many studies have shown that oxidative stress plays a role in the pathogenesis of osteoporosis while several risk factors for osteoporosis such as smoking, hypertension, and diabetes are associated with high levels of oxidative stress [3, 4]. The fall in estrogen levels during the menopausal period leads to a loss of protective effect of estrogen against oxidative stress and reactive oxygen species [5, 6], followed by depletion in antioxidant enzymes in bones [7]. Increased activity of reactive oxygen species (ROS) leads to overexpressions of TNF-*α*, RANKL, and M-CSF which enhance osteoclasts function and induce bone loss [7, 8]. Oxidative stress also suppresses bone formation by inhibiting osteoblast differentiation and decreasing the survival of these cells [9, 10]. The absence of estrogen reduces osteoblastic activity and stimulates osteoclastic activity finally leading to the development of osteoporosis [9].

Virgin coconut oil (VCO) has captured a lot of interest because of its possible role in enhancing body defense against oxidative stress. VCO is different from the ordinary coconut oil as the former contains a lot more biologically active components such as polyphenols, tocopherols, sterols, and squalene [11]. It has been established that the antioxidant activity in VCO is higher than refined coconut oil [12, 13]. VCO has been shown to enhance antioxidant enzymes activity and inhibit the lipid peroxidation in rats [14].

The beneficial effects of VCO have been investigated in various experimental models. Other than having anti-inflammatory, analgesic, and antipyretic effects [15], coconut oil has an antiviral effect whereby it decreases the viral load and increases CD4, CD8 count in HIV patients [16]. The superior moisturizing property of VCO renders it to be more effective in the treatment of atopic dermatitis compared to virgin olive oil [17]. However, to the best of our knowledge, there has not been any work investigating the effect of VCO on bone. In the present study we used ovariectomized rats to simulate postmenopausal osteoporosis, a condition associated with oxidative stress. The aim of the study is to determine the effects of VCO on bone oxidative status in osteoporotic rats by assessing the index of lipid peroxidation and endogenous antioxidant enzymes in the bones.

## 2. Materials and Method

### 2.1. Experimental Animals and Treatment

Thirty-two Sprague-Dawley female rats aged three months old weighing 250–300 g were obtained from the Laboratory Animal Resource Unit, UKM. After being acclimatized for two weeks, they were randomly divided into four groups with eight rats in each group. Two groups of rats were ovariectomised with a group being a negative control and the other one treated with 8% VCO mixed with rat chow. The sham group was sham-operated without removing the ovaries. The baseline group was killed at the beginning of the study. All the rats except the VCO group were fed with normal rat chow diet. The rats were housed two per cage at room temperature with adequate ventilation and normal 12-hour light-dark cycle. They were allowed free access to water and food. The treatment started two weeks postoperatively and lasted for six weeks. This study was approved by the animal ethics committee of UKM (UKM AEC: PP/FAR/2009/NORLIZA/24-FEBRUARY/250-MARCH-2009-JULY-2010.

### 2.2. Preparation of Virgin Coconut Oil Diet

Coconut palm (*Cocos nucifera*) was used to prepare the virgin coconut oil. The virgin coconut oil was prepared based on the method by Nevin and Rajamohan with slight modification [18]. The grated coconut and its neutral water were mixed together to soften the coconut. Then, the coconut mixture was squeezed into viscous slurry until all creamy milk was expelled from the coconut mixture. After that, the creamy coconut milk was kept at room temperature for 48 hours until the fermentation process took place. Three layers were produced as follows: creamy mixture in the upper layer, the virgin coconut oil in the middle, and the water in the lower layer. The oil was gently scooped out and filtered into a container. The 8% VCO diet was prepared by mixing 8 g of VCO with 100 g of rat chow.

### 2.3. Ovariectomy

The rats were anaesthetized, and bilateral ovariectomy was performed for the OVX-groups through ventral approach. The fallopian tubes were tied up before the ovaries were removed. The sham-operated rats underwent the sham procedure whereby the ovaries were exposed and carefully manipulated, but they were left intact [19].

### 2.4. Preparation of Bone Samples

Following six weeks of treatment, the rats were sacrificed using high dose of diethyl ether. The left tibias were cleaned from the adhering muscles and kept at −80°C until they were ready to be tested for malondialdehyde (MDA), superoxide dismutase (SOD), and glutathione peroxidase (GPX).

### 2.5. Measurement of Lipid Peroxidation

The malondialdehyde (MDA) levels in the bones were estimated by using TBARS Assay Kit (Cayman Chemical Company, USA) [20]. The MDA represents the end product of lipid peroxidation. For homogenization of bone sample, the left tibia was ground in a porcelain mortar; 25 mg of bone tissue was weighed and put into 1.5 mL centrifuge tube. It was mixed with 250 mL of RIPA buffer solution with protease inhibitors (EDTA). Then it was sonicated at 40 V for 15 minutes, at 4°C to obtain a homogenate. After that, the homogenate was centrifuged at 1,600 ×g for 10 min (Sigma Laborzentrifugen-3k30, Osterode, Germany). The supernatant was taken and stored at −80°C. The concentration of MDA was measured spectrophotometrically at 540 nm by using Microplate Reader (MBC VERSA max, USA), and the test was performed according to the TBARS Assay Kit instructions.

### 2.6. Measurement of Superoxide Dismutase

First the tibia was homogenized, using this procedure: the bones were perfused with phosphate-buffered saline at PH 7.4 to remove any blood cells or clots. Then 0.25 g of bone was weighed and crushed by using mortar and pestle on ice. The bone tissue was put in 10 mL tube containing 2 mL of 20 mM HEPES buffer (20 mM HEPES buffer pH 7.2, containing 1 mM EGTA, 210 mM Mannitol, and 70 mM sucrose per gram tissue). After that, the tissue was put on ice and homogenized using tissue homogenizer. Next, the homogenized mixture was centrifuged at 1500 ×g for 5 min, 4°C. The supernatant was put in a tube for assaying.

Superoxide dismutase was measured by using Superoxide Dismutase Assay Kit, from Cayman chemical company, USA. SOD was measured spectrophotometrically at 540 nm by using Microplate Reader (MBC VERSA max. USA). The assay was performed according to the Superoxide Dismutase Assay Kit instruction [20].

### 2.7. Measurement of Glutathione Peroxidase

GPX was measured by using Glutathione Peroxidase Activity Assay Kit, from BIOVISION Company, USA [21]. For homogenization of bone sample: 0.1 gram was weighed and put in 10 mL tub on ice. Then 0.2 mL of cold assay buffer was added on ice. After that, the sample was homogenized by using OMNI BEAD RUPTOR 24, for 50 seconds. Then it was put on ice till the temperature decreases. The mixture was centrifuged at 10,000 xg for 15 minutes at 4°C by using Microcentrifuge 22 R (Beckman Coulter bench top refrigerated micro centrifuge, Germany). Finally, the supernatant was collected in eppendorf tube for assay. The GPX was estimated by measuring the optical density (OD) of the samples at 340 nm by using Micro Plate Reader (MBC VERSA max, USA). The assay was performed according to the Glutathione Peroxidase Activity Assay Kit instruction.

## 3. Results

### 3.1. Bone Lipid Peroxidation (TBARS)

**Figure 1:**
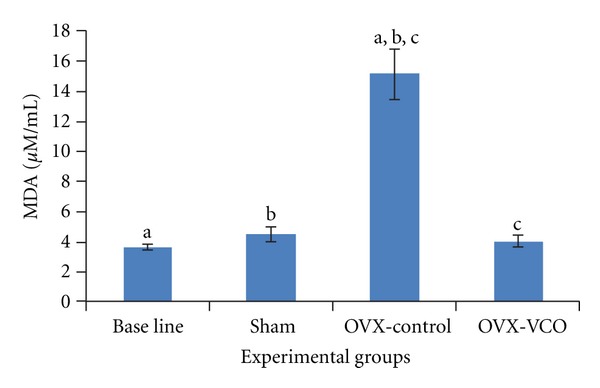
This figure shows MDA levels in different groups of rats. Same letters indicate significant difference-between groups at *P* < 0.05. OVX-VCO group (ovariectomised-received virgin coconut oil group). OVX-control group (ovariectomised control group). Sham group (sham-operated group).

 There was a significant decrease in the concentration of MDA (*P* < 0.05) in the bone of OVX-VCO group compared to OVX-control group. In addition, MDA level was significantly increased in the bone of OVX-control group compared to baseline and sham groups (Figure 1).

### 3.2. Glutathione Peroxidase

**Figure 2:**
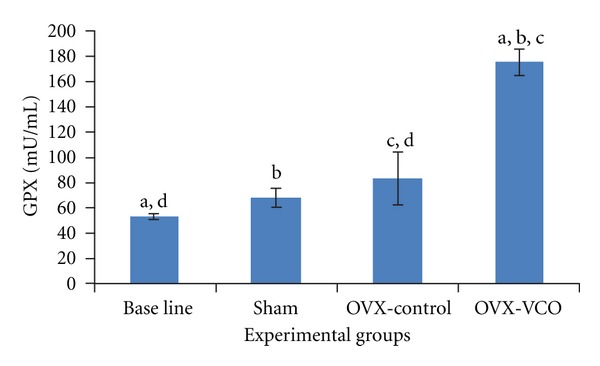
This figure shows GPX levels in different groups of rats. Same letters indicate a significant difference between groups at *P* < 0.05. OVX-VCO group (ovariectomised-received virgin coconut oil group). OVX-control group (ovariectomised-control group). Sham group (sham-operated group).

There was a marked improvement in the antioxidant status of the bone in OVX-VCO group which was reflected by a significant increase in the concentration of GPX (*P* < 0.05) compared to OVX-control group. In addition, the GPX level was significantly increased in OVX-control group compared to baseline group (*P* < 0.05) (Figure 2).

### 3.3. Superoxide Dismutase

**Figure 3:**
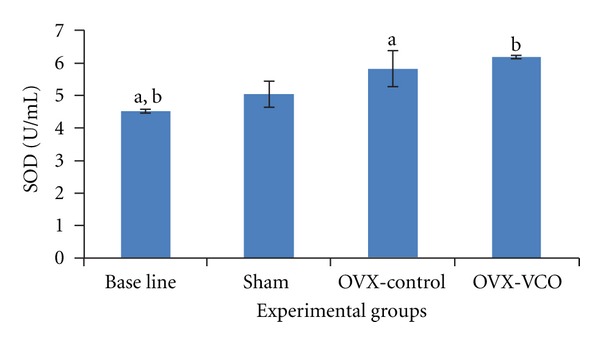
This figure shows SOD levels in different groups of rats. Same letters indicate a significant difference between groups at *P* < 0.05. OVX-VCO group (Ovariectomised-received virgin coconut oil group). OVX-control group (ovariectomised-control group). Sham group (sham-operated group).

 The SOD concentration was increased in the bone of OVX-VCO group compared to OVX-control group, but the change was not statistically significant. The SOD level was significantly increased in OVX-control group compared to baseline group (*P* < 0.05) (Figure 3).

### 3.4. Statistical Analysis

SPSS version 19 was used for analysis of data. Data was tested for normality by using Kolmogorov-Smirnov normality test. Normally distributed data was analyzed using one-way ANOVA. The results were presented as means ± SEM.

## 4. Discussion

Reduction in estrogen level is the major cause of bone loss in postmenopausal osteoporosis [22]. The ovariectomised rats are the recommended animal model for investigating preclinical therapies for postmenopausal osteoporosis [23], since the bone changes in ovariectomy and postmenopausal state are similar. The reduction in endogenous estrogen levels in both situations causes an increase in the bone turnover which leads to enhanced bone loss and a decrease in the bone mineral density [24, 25].

Postmenopausal osteoporosis is associated with oxidative stress and inhibition of the antioxidant defense system [22], resulting in the imbalance between osteoblast and osteoclast activities. Previously, we demonstrated that virgin coconut oil significantly improved the bone histomorphometric parameters, including the trabecular number, trabecular thickness, and trabecular separation in ovariectomised rats (unpublished data). The positive findings in the histomorphometric study in the bone directed us to further investigate the effect of VCO on oxidative status in the bone of osteoporotic rat model as an attempt to understand the role of VCO in enhancement of the body defense system against oxidative stress and free radicals.

The results of the present study showed significant improvement in the bone antioxidant status after VCO supplementation by a significant increase in the levels of glutathione peroxidase in OVX-VCO group compared to OVX-control group, with an increased trend of SOD levels. The positive effect on the antioxidant enzymes was supported by a low level of MDA in OVX-VCO group. In the same way, the significant increase in the levels of GPX and SOD in the ovariectomised-control rats represented the endogenous release of antioxidant enzymes, in response to oxidative stress and the high free radical activity in bone. Nonetheless, this elevation in the antioxidant enzymes was unable to suppress the lipid peroxidation which explained the significantly high level of MDA in OVX-control group.

Our results showed some similarities with the studies involving postmenopausal osteoporotic women, whereby the antioxidant parameters such as the total antioxidant capacity (TAC), plasma activity of SOD, catalase, and glutathione reductase were significantly increased compared to normal women, but this physiological elevation in the antioxidants was not enough to prevent the development of osteoporosis [26, 27]. In contrast, several studies indicated that the activity of GPX in the plasma was significantly reduced in postmenopausal women compared to healthy women [28, 29]. Glutathione peroxidase has an important role in reducing lipid hydroperoxide, which breaks down the oxidation chain and suppresses the free radicals release [30]. Dreher et al. [31] reported that the reduction in GPX expression could interfere with osteoblast functions and enhance the bone loss leading to osteoporosis. In addition, GPX expression by osteoblast was increased in response to oxidative stress [32].

The role of VCO in preventing oxidative stress was also manifested in other organs as well. VCO was shown to have superior suppressive effect on microsomal lipid peroxidation compared to copra oil and groundnut oil [14]. VCO stimulated the antioxidant enzymes activity and decreased the MDA and glutathione levels in healing wounds. This inhibition in lipid peroxidation promoted fibroblast proliferation, neovascularization, and healing process [33]. In addition, blending of VCO with groundnut oil or olive oil was proven to be effective in inhibiting LDL oxidation, and stimulating the activity of hepatic antioxidant enzymes [34].

The antioxidant activity of VCO is due to the high composition of polyphenol compounds in the oil [11, 14, 35]. Marina et al. estimated the total phenolic content of VCO to be in the range of 7.78–29.18 mg GAE/100 g oil, which is significantly higher than the refined, bleached, and deodorized coconut oil [35]. The major polyphenols in VCO are ferulic acid and p-coumaric acid [35]. Seneviratne and Dissanayake (2008) also detected the presence of ferulic acid, p-coumaricacid, and caffeic acid in the commercial and traditional VCO [36]. Polyphenols are stronger as antioxidants than vitamins C and E *in vitro* on the molar basis [37].

The antioxidant properties of ferulic acid have been established. Ferulic acid belongs to phenoxy carboxylic acid family [38]. Toda et al. [39] have proven that ferulic acid has the ability to scavenge the superoxide radical and suppress the lipid peroxidation induced by superoxide anion. Superoxide radicals can enhance bone resorption by degrading matrix proteins, making the bones weak and easily digested by enzymes [5]. Ries et al. [40] have reported that a superoxide radical scavenger such as Desferal-manganese complex can reduce superoxide production and decrease bone resorption by osteoclast.

 The effects of ferulic acid and superoxide dismutase as antioxidants were equal in magnitude, and this characteristic made it superior to caffeic acid and p-coumaricacid as an antioxidant [39]. In addition, the effect of ferulic acid as inhibitor of lipid peroxidation was similar to the effect of *α*-tocopherol [39]. Castelluccio et al. [41] reported that ferulic acid was more potent as an antioxidant against LDL oxidation than ascorbic acid. It seems that VCO derives most of its effects from the free-radical scavenging and antioxidant properties of ferulic acid.

The antioxidant power of ferulic acid is due to its ability to effectively end the terminal radical chain reactions, since any free radical colliding with ferulic acid molecule can easily extract a hydrogen atom from the phenolic hydroxyl group to form a phenoxy radical which is considered a highly stable compound [38]. This phenoxy radical is unable to initiate or propagate the reactive chain reaction. This stability belongs to easy formation and lack of reactivity of phenoxy radical. Moreover, there is extended conjugation in the unsaturated side chain of phenoxy radical, and the unpaired electron may not be attached to oxygen atom, but it can move throughout the entire molecule [38].

There are few studies that investigate the effects of phenolic acids on bone loss. Sassa et al. [42] reported that ferulic acid enhanced bone remodeling process by stimulating osteoblasts to compensate for the bone loss by osteoclasts, and ferulic acid raised serum level of estrogen, progesterone in postmenopausal osteoporotic rat model. Zych et al. [43] showed that caffeic acid and p-coumaricacid increase serum estrogen levels in estrogen deficiency rat model. The authors suggested that phenolic acids such as caffeic acid may affect the metabolic pathway which regulates the extraovarian estrogen release [43]. Folwarczna et al. [44] investigated the effects of phenolic acids on bone loss in postmenopausal osteoporotic rat model, and they reported that p-coumaricacid had a positive effect on the bone mass/body-mass ratio and bone mineral mass/body-mass in bone. On the other hand, phenolic acids have no effect on bone mineral mass/bone mass in the bone of ovariectomised rats.

## 5. Conclusion

The imbalance between oxidative stress and antioxidant agents leads to enhancement of osteoclast activity and inhibition of osteoblast activity. Diet rich with antioxidants is considered as a novel therapeutic agent in prevention and treatment of postmenopausal osteoporosis. VCO can prevent lipid peroxidation and increase the antioxidant enzymes in the osteoporotic rat model. Therefore, supplementation of antioxidant-enriched diet as virgin coconut oil may shed light on the development of new alternative therapy for postmenopausal osteoporosis and prevention of fractures. However, further studies are necessary in order to obtain a more complete evaluation of the therapeutic potential and safety profile of the oil.
